# How is the Topic of Intersex Athletes in Elite Sports Positioned in Academic Literature Between January 2000 and July 2022? A Systematic Review

**DOI:** 10.1186/s40798-022-00520-0

**Published:** 2022-10-20

**Authors:** Marisa Jensen, Jörg Schorer, Irene R. Faber

**Affiliations:** 1 Institute of Sport Science, Carl Von Ossietzky University, Ammerländer Heerstraße 114-118, 26129 Oldenburg, Germany; 2 Research Centre Human Movement and Education, Windesheim University of Applied Sciences, Zwolle, The Netherlands

**Keywords:** Intersex variations, Elite sports, DSD, Hyperandrogenism

## Abstract

**Background:**

Within the topic of intersex athletes in elite sports, science has become a decisive factor in decision- and policy-making. However, in the academic literature approaches to this topic vary. An overview of these approaches is proposed to provide better insight into relevant aspects and underlying values and may serve as a starting point on the path toward a solid solution of the question of categorization of intersex athletes in elite sporting competition.

**Objective:**

This systematic review aims to discover how the topic of intersex elite athletes is positioned in the academic literature from January 2000 to July 2022 from a neutral perspective.

**Methods:**

A comprehensive search in eleven databases using the search terms [intersex* and sport*] yielded 87 articles. A qualitative content analysis was conducted to find all authors’ statements including perspectives on intersex athletes and proposals for solutions. Underlying values were extracted and connected to each other during axial coding.

**Results:**

The results provide an overview of the sometimes-contradictory perspectives toward intersex elite athletes and proposals for solutions. Three core values were distilled: social justice for intersex elite athletes, competition fairness, and evidence-based practice. The authors’ statements disclose an interaction/conflict between social justice and competition fairness.

**Conclusions:**

The results raise an important discussion on the role of science within the topic of intersex elite athletes. A multidisciplinary approach including scientists and other experts is suggested to find an appropriate solution. Additionally, more awareness on intersex variations is needed for a better overall understanding and to ensure a respectful approach for everyone involved.

## Key Points

- This systematic review provides an overview of the perspectives on categorization of intersex athletes in elite sports and proposals for solution of the issues involved based on authors’ statements in articles published between January 2000 and July 2022.
- The perspectives point out criticism concerning the binary categorization in elite sports, sports regulations, and a research gap regarding the topic of intersex athletes and elite sporting performance. The proposals for solutions include discussions on how to enable the binary division of all athletes and alternative categorizations for elite sports. These results can be used as a starting point for additional research on the topic of intersex athletes and further, for finding an adequate solution.
- The values underlying the authors’ statements indicate the topic’s complexity and reveal the conflict between the approaches for creating social justice while maintaining competition fairness.

## Introduction

People who can be characterized as “intersex” have differences in sexual development that encompass natural variations in typically male or female sexual characteristics. These differences are not consistent with common binary notions of male and female bodies. This can be a complex topic for society that raises many questions and much discussion [1–3]. A lack of understanding about intersex variations has caused problems, especially in areas where the binary construct of sex (i.e., male and female) is obvious. In modern-day sports, especially women’s sports, the topic of intersex athletes has developed into one of the most contentious issues [4]. Different stakeholders have commented on, criticized, and questioned the topic for years [5–7]. Still, what is missing is an approach free of discrimination, humiliation, and exclusion, to address individuals that are somewhere between the distinct categories provided by sports governing bodies. Science^1^ has become an evident deciding factor in the inclusion of intersex individuals/athletes in sports. Indeed, sports governing bodies always consult scientists to support decision- and policy-making. However, approaches toward intersex athletes in elite sports within science appear to vary. In the following, an overview of these approaches is examined to provide better insight into the aspects and underlying values relevant to intersex athletes from the academic perspective and serve as a starting point to establish a solid solution in the future.

There is a general usage of a binary sex construct in most societies. For clarification, “sex” describes a person’s biological status, whereas “gender” refers to a socially constructed concept that describes an individual’s fundamental sense of belonging to specific sexes [8, 9]. People differing from this binary sex construct of male and female are referred to as “intersex individuals” [10]. The term is used to describe an ambiguity in one of the factors that typically determine sex, such as genetics, genitalia, and hormones.^2^

Intersex variations are suggested to exist from the beginning of humankind and were formerly titled “hermaphrodite” [12]. The geneticist Richard Goldschmidt coined the term “intersexuality” in 1915. Still, one century later, the knowledge about it among society is deficient. This is mainly because intersex individuals were secretly “adjusted” straight after birth for years, following a lifelong prescribed medical treatment [12–15]. In general, intersex variations can appear in several different forms, sometimes undetected at birth. Experts’ opinions differ on prevalence percentages, suggesting that it appears between 0.02% and nearly 2% [13, 16–19].

The major reason intersex variations cause difficulties in sports, especially in elite women’s sports, is the role of androgens, a class of hormones responsible for developing and maintaining masculine characteristics [20, 21]. The major androgen circulating in human blood is testosterone [22]. To what extent testosterone, either endogenous or exogenous, enhances sporting performance within the female category has been a contested topic in the past years [23–25]. Because some intersex variations entail hyperandrogenism, a trait in which the individual produces high levels of androgen (e.g., testosterone), sports governing bodies are worried about potential advantages over other athletes competing in the women’s category. As most female sports, like male sports, evolved to highly competitive environments with medals and financial incentives at stake during the twentieth century and beyond, it is no surprise that historical events and “suspicious” cases have shaped the approach to inclusion of intersex athletes in elite sports.

At the beginning of sports, participation was restricted to men only. Since women were allowed to enter the Olympics in 1900, the division of athletes has been following a dual construct: male and female [26]. The main reason for this is that, on average, men are stronger, faster, and bigger than women. Historically, suspicions of sex fraud in women’s sports resulted in discriminatory and humiliating sex verification tests [18]. These verification tests failed to uncover men masquerading as women. Instead, they detected several athletes with intersex variations competing in the female category. Not knowing how to cope with such diversity, the athletes in question were excluded from competitions [18].

According to Bassett et al. [27], sex verification tests were introduced by World Athletics, formerly known as the International Association of Athletics Federation (IAAF), in 1950. In the beginning, the so-called nude parades focused solely on the outer appearance, forcing athletes that wanted to compete in the female category to undress and parade naked in front of gynecologists [24, 28, 29]. Due to the resistance to these procedures and technological advances, genetic checks were first used in the Grenoble Winter Olympic Games and the Mexico Summer Olympics in 1968 [18]. In contrast to the prior tests, the concern had moved away from the outer appearance of the athletes. Instead, the Barr body tests looked for the second X chromosome, assuming that a male usually constitutes an XY and a female an XX chromosome pattern. Further, the possession of a Y chromosome was believed to produce superior athletic ability [18]. In 1992, the International Olympic Committee (IOC) replaced these tests with chromosome tests that used DNA polymerase chain reaction. Still, the decision of whether one was allowed to compete in the female category depended on the absence of a Y chromosome. In the same year, World Athletics decided to abandon mandatory mass testing and only compelled sex verification in particular cases of doubt [18, 28]. In 1999, the IOC also abandoned the mass sex testing of athletes competing in the female category, starting with the Olympic Games in Sydney in 2000. What remained were “suspicion-based” medical examinations for questionable cases [30, 31].

Nevertheless, this did not solve the topic of intersex athletes in elite sports. There have been years of struggle for athletes and governing bodies. World Athletics still struggles, with fitting the seemingly complex sex, provided by nature into the binary categorization created by society. The topic arose in the media once the South African middle-distance runner Caster Semenya was publicly suspected during World Championships in Berlin in 2009 [32, 33]. Her physical appearance was perceived to contradict the standard norms of femininity [34]. Reacting to Semenya, the IOC and World Athletics established a regulation that came into effect on May 1, 2011. This regulation stipulated athletes with high androgen levels wanting to compete in the female category must lower their androgen level to below 10 nmol/L [34]. These regulations were suspended by the Court of Arbitration for Sport (CAS) in 2015 due to insufficient scientific data [35].

In 2017, Bermon and Garnier [36] published a study presenting a significant advantage of female athletes with high androgen levels over other female athletes in three running disciplines: 400 m sprint, 400 m hurdles, and 800 m sprint. The research was highly contested, so the authors decided to upload a correction in 2021 [37]. In April 2018, World Athletics released their latest approach, only addressing athletes who possess a 46 XY DSD and a circulating testosterone level of 5 nmol/L or above. The term “46 XY DSD” describes differences in sexual development (DSD) with an XY chromosome pattern. Additionally, the regulations are restricted to events from 400 m to one mile. It allows the aforesaid elite athletes to compete in the female category if they undergo a hormonal treatment to lower their androgen level to below 5 nmol/L or prove they possess complete androgen insensitivity [38]. Although they might meet the expectations of fair play, the Fundamental Principles of Olympism also attribute every athlete the right to compete without discrimination of any kind. This raises the question of whether a medical treatment to change an individual’s natural hormone balance is justifiable. Caster Semenya filed a challenge against these new regulations before the Court of Arbitration for Sport (CAS) which was rejected on May 1, 2019, because she could not prove the new regulations’ invalidity [39]. On February 18, 2021 Caster Semenya appealed to the European Court of Human rights (ECHR) [40]; the case is currently pending.

It is clear that intersex variations, specifically in elite women’s sports, are a highly complex topic that needs careful consideration. As mentioned before, science is a deciding factor as scientific evidence is used to support decision- and policy-making. From a neutral perspective, this review aims to summarize the approaches to intersex elite athletes reported in the academic literature in the twenty-first century. The examination will help us understand the relevant aspects and underlying values from the academic perspective. It will also aid the development of an approach to intersex elite athletes free of discrimination, humiliation, and exclusion.

## Methods

### Systematic Search

This systematic review followed the guidelines of the Preferred Reporting Items for Systematic Reviews and Meta-Analyses (PRISMA) statement [41]. The systematic search covered the period from January 2000 to July 2022 (Date of search: August 8, 2022). Relevant articles were identified through eleven databases, particularly for sports. The databases were PubMed, Web of Science, SPONET, SPORTDiscus, SURF, BASE, Scopus, ScienceDirect, JSTOR, ProQuest, and Darwin Correspondence Project. The terms used for the systematic search were (intersex* AND sport*). No limitations were used during searches within all databases except for the time frame. Additionally, a manual search was conducted on Google Scholar.

### Article Selection

After the systematic search was conducted, relevant articles were selected according to inclusion and exclusion criteria. Articles were included if (1) they broach the topic of intersex variations, (2) they focus on elite sports, (3) the authors express an approach toward intersex athletes in elite sports, and (4) they are written in English or German. In turn, references were excluded if (1) they focus only on the differences between men and women in elite sports, (2) they concern mass sports, and (3) they were published before 2000. The sifting was carried out in two stages, as recommended by Boland et al. [42]. The retrieved references were screened by title and abstract in relation to the inclusion and exclusion criteria. This was conducted independently by two investigators (MJ and LS). A third expert (IF) was consulted in case of conflict to reach consensus.

### Data Synthesis

First, for overview purposes, relevant descriptive data of all studies were systematically extracted and transferred into a table (i.e., authors, year of publication, title, journal, and country of the corresponding author’s affiliation). Second, an inductive thematic analysis of the content was conducted, following the six phases of analysis as proposed by Braun and Clarke [43]. As a first step, the articles were scoured for their information on approaches to address intersex athletes in elite sports. After an initial coding of interesting features throughout the articles, the different codes were further sorted into potential themes. After reviewing the themes, two main categories were established: (1) perspectives on intersex athletes and (2) proposals for solutions, that each cover the four subthemes: (1) the sex construct, (2) sex verification in elite sports, (3) fair play and intersex athletes, and (4) information and knowledge. As such, each of the two categories is described while including citations of the authors’ statements for each of the four themes. As a next step, the information found was screened to reveal the underlying values. Therefore, the author’s statements were considered in detail, including the context within which they were made. These values were independently extracted by two investigators (MJ and IF). In case of conflicts, a consensus was reached through discussion in the research group. Subsequently, axial coding was conducted by all authors (MJ, JS, IF) in a peer debriefing (three separate sessions) to find the connections, interactions, and coherence between the values and the topic of intersex elite athletes.

## Results

### Systematic Search

**Fig. 1:**
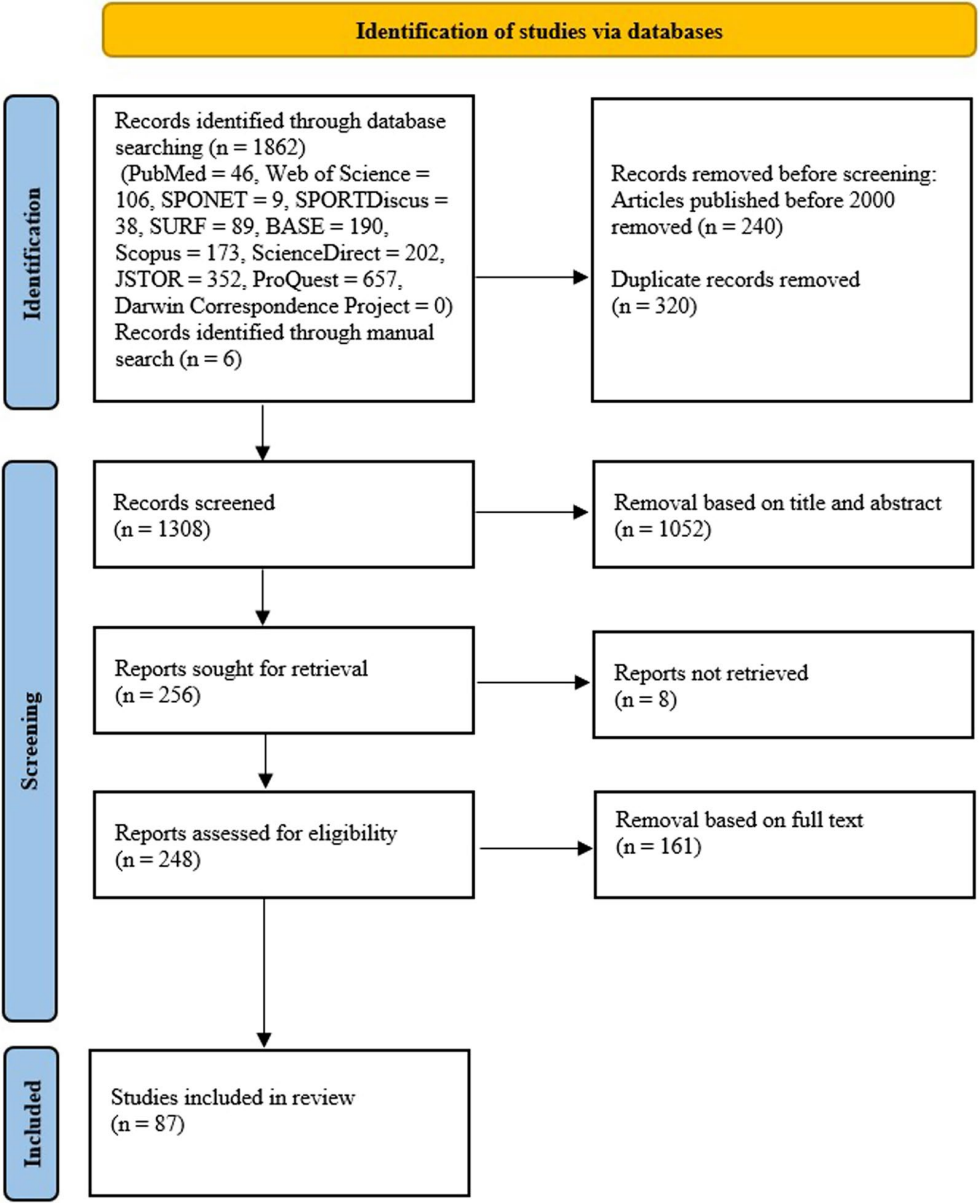
Flowchart of systematic search

The systematic search obtained a total of 1862 references (Fig. 1). A manual search on Google Scholar yielded six additional articles. After the removal of all references published before 2000 (*n* = 240), the number was reduced to 1628. The removal of duplicates (*n* = 320) and the exclusion of title and abstract according to the eligibility criteria (*n* = 1052) further downsized the number to 256. Articles that were not possible to access (*n* = 8) were excluded from further examination. The main reason for exclusion after assessing full texts (*n* = 161) was that the articles did not include an approach to intersex elite athletes and/or the allied regulations. Combined, this resulted in a total of 87 articles included in the review.

### Descriptive Results

**Fig. 2:**
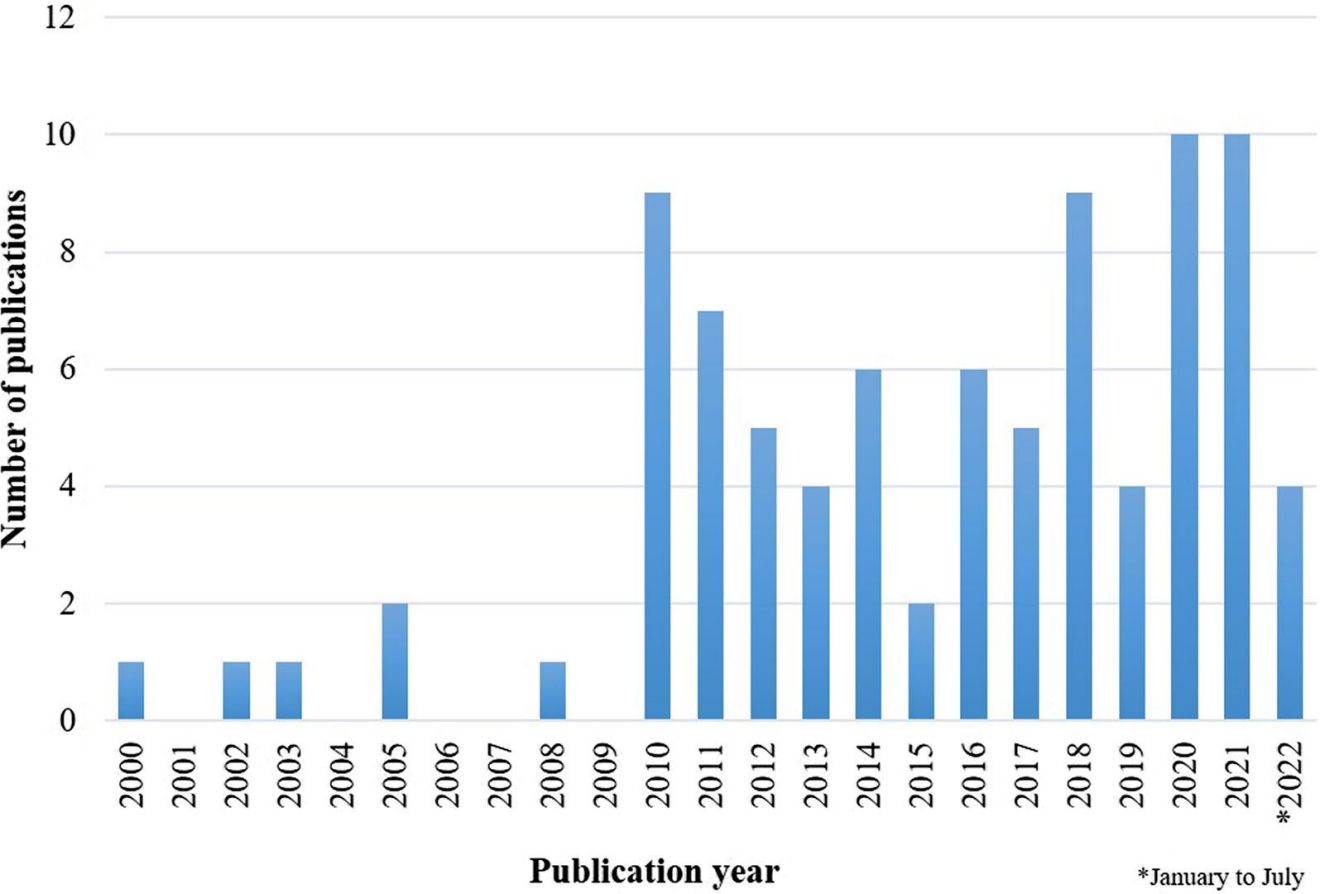
Histogram article distribution

The descriptive data of the included articles are presented in Table 1. Figure 2 shows the number of articles published per year. Most articles have been published since 2010 (*n* = 81) with the highest increase in 2020 and 2021 (*n* = 11); the oldest included article was from 2000. In 37 out of the 87 articles, the corresponding author was affiliated with an institution in the USA. The second most common country was Great Britain (*n* = 15), third most common was Germany (*n* = 6). Fourth were Sweden and Switzerland (*n* = 4) followed by India, Canada, and South Africa (*n* = 3) and Australia, Spain, and Italy (*n* = 2). The least frequent were France, Poland, Norway, Finland, the Czech Republic, and the Netherlands with one each. A closer look at the journals indicates that 79 out of 87 articles have been published in peer-reviewed journals.

**Table 1:** Characteristics of studies included in the systematic review in chronological order (publication year)

Author	Year	Country	Perspective/solution	Theme	Statement
Elsas et al. [44]	2000	USA	Perspective	Sex verification	Sex verification is ethically indefensible
			Solution	Information and knowledge	Educate about sex diversity
Dickinson et al. [45]	2002	USA	Perspective	Sex verification	Sex verification is ethically indefensible
				Information and knowledge	High androgen levels do not always provide an athletic advantage
			Solution	Sex verification	Abolish sex verification
Wackwitz [46]	2003	USA	Perspective	The sex construct	Sex is not binary
				Sex verification	Sex verification is ethically indefensible
Carlson [47]	2005	USA	Perspective	The sex construct	Sex is not binary
				Sex verification	Sex verification is ethically indefensible
				Information and knowledge	High androgen levels do not always provide an athletic advantage
Reeser [48]	2005	USA	Perspective	Sex verification	Sex verification is ethically indefensible
				Information and knowledge	High androgen levels do not always provide an athletic advantage
Ritchie et al. [6]	2008	GB	Perspective	Sex verification	Sex verification is ethically indefensible
				Information and knowledge	High androgen levels do not always provide an athletic advantage
Amy-Chinn [32]	2010	GB	Perspective	The sex construct	Sex is not binary
				Information and knowledge	High androgen levels do not always provide an athletic advantage
Camporesi and Maugeri [49]	2010	IT	Perspective	The sex construct	Sex is not binary
				Fair play and intersex athletes	Excluding intersex athletes from elite sporting competition is unfair
				Information and knowledge	High androgen levels do not always provide an athletic advantage
			Solution	Fair play and intersex athletes	Do not use a third category to separate sporting competition
				Information and knowledge	Include other specialists to find a solution
Dreger [50]	2010	USA	Perspective	The sex construct	Sex is not binary
				Information and knowledge	Information on intersex variations/androgens is lacking
			Solution	Fair play and intersex athletes	Use self-identification as a criterion to separate sporting competition
Hercher [51]	2010	USA	Perspective	Sex verification	Gender cannot be identified through simple test
					The athlete’s privacy must be ensured
				Fair play and intersex athletes	High androgen levels are one of many athletic advantages
				Information and knowledge	High androgen levels do not always provide an athletic advantage
			Solution	The sex construct	Do not abolish sex segregation in sporting competition
				Fair play and intersex athletes	Intersex athletes should limit androgens to compete in the female category
Merck [52]	2010	GB	Perspective	Sex verification	The athlete’s privacy must be ensured
				Information and knowledge	High androgen levels do not always provide an athletic advantage
			Solution	The sex construct	Abolish sex segregation in sporting competition
Nerva [53]	2010	GB	Perspective	Information and knowledge	Information on intersex variations/androgens is lacking
Saleem [54]	2010	USA	Perspective	Sex verification	Sex verification is ethically indefensible
					The athlete’s privacy must be ensured
			Solution	Sex verification	Abolish sex verification
Tucker and Collins [55]	2010	ZA	Perspective	Information and knowledge	Information on intersex variations/androgens is lacking
Wonkam et al. [56]	2010	ZA	Perspective	Sex verification	Sex verification can cause great harm
					The athlete’s privacy must be ensured
				Information and knowledge	High androgen levels do not always provide an athletic advantage
			Solution	Sex verification	Set a clear policy on sex verification
				Information and knowledge	Include other specialists to find a solution
Vannini and Fornssler [57]	2011	CA	Perspective	The sex construct	Sex is not binary
Foddy and Savulescu [58]	2011	GB	Perspective	The sex construct	Sex is not binary
					Sex segregation in sporting competition is unjust
				Fair play and intersex athletes	Excluding intersex athletes from elite sporting competition is unfair
			Solution	The sex construct	Abolish sex segregation in sporting competition
				Fair play and intersex athletes	Intersex athletes should limit androgens to compete in female’s category
Wiesemann [59]	2011	DE	Perspective	Sex verification	Sex verification is ethically indefensible
					Sex verification can cause great harm
				Information and knowledge	High androgen levels do not always provide an athletic advantage
			Solution	Sex verification	Abolish sex verification
Schultz [60]	2011	USA	Perspective	Fair play and intersex athletes	High androgen levels are one of many athletic advantages
				Information and knowledge	Information on intersex variations/androgens is lacking
			Solution	Information and knowledge	Include other specialists to find a solution
Adair [28]	2011	USA	Perspective	Sex verification	Sex verification is ethically indefensible
					Sex verification supports stereotypes
					The athlete’s privacy must be ensured
				Information and knowledge	High androgen levels do not always provide an athletic advantage
			Solution	The sex construct	Abolish sex segregation in sporting competition
				Sex verification	Set a clear policy on sex verification
				Information and knowledge	Include other specialists to find a solution
Ballantyne et al. [61]	2011	NL	Perspective	Sex verification	The athlete’s privacy must be ensured
				Information and knowledge	High androgen levels do not always provide an athletic advantage
			Solution	Fair play and intersex athletes	Intersex athletes should limit androgens to compete in the female category
				Information and knowledge	Include other specialists to find a solution
Pierson [62]	2011	USA	Perspective	Sex verification	Privacy is a sacrifice when competing in elite sports
Glazer [23]	2012	USA	Perspective	Fair play and intersex athletes	The regulations on hyperandrogenism from 2011 are ethically debatable
					High androgen levels are one of many athletic advantages
				Information and knowledge	Information on intersex variations/androgens is lacking
Vilain and Sánchez [63]	2012	USA	Perspective	Fair play and intersex athletes	The regulations on hyperandrogenism from 2011 are ethically debatable
				Information and knowledge	Information on intersex variations/androgens is lacking
					High androgen levels do not always provide an athletic advantage
			Solution	Fair play and intersex athletes	Set new criteria to separate sporting competition
Viloria and Martinez-Patino [64]	2012	USA	Perspective	Fair play and intersex athletes	The regulations on hyperandrogenism from 2011 are ethically debatable
				Information and knowledge	Information on the treatment set by IOC/World Athletics in 2011 is lacking
			Solution	Fair play and intersex athletes	Abolish the 2011 regulations on hyperandrogenism
Wahlert and Fiester [65]	2012	USA	Perspective	Information and knowledge	Information on intersex variations/androgens is lacking
Wiederkehr [66]	2012	DE	Perspective	The sex construct	Sex is not binary
			Solution	Fair play and intersex athletes	Do not use a third category to separate sporting competition
Cooky and Dworkin [67]	2013	USA	Perspective	Fair play and intersex athletes	The regulations on hyperandrogenism from 2011 are ethically debatable
				The sex construct	Sex is not binary
			Solution	Fair play and intersex athletes	Set new criteria to separate sporting competition
					Treat all advantages in sporting competition equally
				Sex verification	Abolish sex verification
Sánchez et al. [68]	2013	USA	Perspective	Fair play and intersex athletes	High androgen levels are one of many athletic advantages
			Solution	Fair play and intersex athletes	Do not use a third category to separate sporting competition
Gandert et al. [69]	2013	USA	Perspective	Information and knowledge	Information on intersex variations/androgens is lacking
Dabholkar [70]	2013	IN	Perspective	Fair play and intersex athletes	The regulations on hyperandrogenism from 2011 are ethically debatable
			Solution	Fair play and intersex athletes	Abolish the 2011 regulations on hyperandrogenism
Ferguson-Smith and Bavington [71]	2014	GB	Perspective	Fair play and intersex athletes	The regulations on hyperandrogenism from 2011 are ethically debatable
				Information and knowledge	Information on intersex variations/androgens is lacking
					Information on the treatment set by IOC/World Athletics in 2011 is lacking
					High androgen levels do not always provide an athletic advantage
			Solution	Fair play and intersex athletes	Abolish the 2011 regulations on hyperandrogenism
Teetzel [72]	2014	CA	Perspective	Sex verification	The athlete’s privacy must be ensured
				Fair play and intersex athletes	The regulations on hyperandrogenism from 2011 are ethically debatable
			Solution	Fair play and intersex athletes	Set new criteria to separate sporting competition
					Include women in the debate
Zehnder [26]	2014	CH	Perspective	The sex construct	Sex is not binary
			Solution	The sex construct	Abolish sex segregation in sporting competition
				Fair play and intersex athletes	Do not use a third category to separate sporting competition
					Set new criteria to separate sporting competition
Henne [73]	2014	CA	Perspective	The sex construct	Sex is not binary
				Fair play and intersex athletes	The regulations on hyperandrogenism from 2011 are ethically debatable
					High androgen levels are one of many athletic advantages
Jakubowska [74]	2014	PL	Perspective	The sex construct	Sex is not binary
				Fair play and intersex athletes	The regulations on hyperandrogenism from 2011 are ethically debatable
Pieper [75]	2014	USA	Perspective	Fair play and intersex athletes	The regulations on hyperandrogenism from 2011 are ethically debatable
Blithe and Hanchey [76]	2015	USA	Perspective	The sex construct	Sex is not binary
				Sex verification	Sex verification is ethically inconsistent
Sonksen et al. [77]	2015	GB	Perspective	Fair play and intersex athletes	The regulations on hyperandrogenism from 2011 are ethically debatable
			Solution	Fair play and intersex athletes	Abolish the 2011 regulations on hyperandrogenism
Buzuvis [78]	2016	GB	Perspective	The sex construct	Sex is not binary
				Fair play and intersex athletes	Diversity is what makes sports interesting
					High androgen levels are one of many athletic advantages
			Solution	Fair play and intersex athletes	Use self-identification as a criterion to separate sporting competition
Gleaves and Lehrbach [79]	2016	USA	Perspective	Fair play and intersex athletes	Excluding intersex athletes from elite sporting competition is unfair
			Solution	The sex construct	Abolish sex segregation in sporting competition
Müller [80]	2016	DE	Perspective	The sex construct	Sex is not binary
Newbould [5]	2016	GB	Perspective	The sex construct	Sex is not binary
				Fair play and intersex athletes	The regulations on hyperandrogenism from 2011 are ethically debatable
			Solution	Fair play and intersex athletes	Do not use testosterone as a criterion to separate sporting competition
					Do not use biological parameters as criteria for separating sporting competition
Pitsiladis et al. [81]	2016	GB	Perspective	Information and knowledge	Information on intersex variations/androgens is lacking
Lovett [82]	2016	USA	Solution	Information and knowledge	Include other specialists to find a solution
Auchus [25]	2017	USA	Perspective	Sex verification	The athlete’s privacy must be ensured
				Information and knowledge	High androgen levels provide an athletic advantage
			Solution	Fair play and intersex athletes	Intersex athletes should limit androgens to compete in female’s category
					Do not use self-identification as a criterion to separate sporting competition
Pielke [83]	2017	USA	Perspective	The sex construct	Sex is not binary
				Fair play and intersex athletes	High androgen levels are one of many athletic advantages
			Solution	Fair play and intersex athletes	Do not use self-identification as a criterion to separate sporting competition
Katz and Luckinbill [84]	2017	USA	Solution	Fair play and intersex athletes	Use self-identification as a criterion to separate sporting competition
Ingthorsson [85]	2017	SE	Perspective	Fair play and intersex athletes	The regulations on hyperandrogenism from 2011 are ethically debatable
					Diversity is what makes sports interesting
					High androgen levels are one of many athletic advantages
				Information and knowledge	Information on intersex variations/androgens is lacking
			Solution	Fair play and intersex athletes	Do not use a third category to separate sporting competition
Davis and Preves [86]	2017	USA	Perspective	The sex construct	Sex is not binary
Ljungqvist [87]	2018	SE	Perspective	Fair play and intersex athletes	A new rule for intersex athletes in elite sports is necessary
Karkazis and Jordan-Young [7]	2018	USA	Perspective	Fair play and intersex athletes	The regulations on hyperandrogenism from 2011 are ethically debatable
Fields [88]	2018	USA	Perspective	Sex verification	The athlete’s privacy must be ensured
				Information and knowledge	Information on intersex variations/androgens is lacking
			Solution	Sex verification	Set a clear policy on sex verification
Linghede [89]	2018	SE	Perspective	The sex construct	Sex is not binary
Virgilii [90]	2018	IT	Solution	Fair play and intersex athletes	Use categories based on weight and height to separate sporting competition
Harper et al. [4]	2018	CH	Perspective	The sex construct	Binary categories for elite sporting competition are appropriate
				Information and knowledge	Information on intersex variations/androgens is lacking
			Solution	Fair play and intersex athletes	Use an athletic gender
					The World Athletics’ 2018 Eligibility Rules are currently the best method
Jakob [91]	2018	DE	Perspective	The sex construct	Binary categories for elite sporting competition are appropriate
				Information and knowledge	Information on intersex variations/androgens is lacking
			Solution	Fair play and intersex athletes	Do not use a third category to separate sporting competition
					Do not use testosterone as a criterion to separate sporting competition
Karkazis and Carpenter [92]	2018	USA	Perspective	Fair play and intersex athletes	The World Athletics’ 2018 Eligibility Rules are ethically debatable
				Information and knowledge	Information on intersex variations/androgens is lacking
Harper et al. [93]	2018	GB	Perspective	The sex construct	Sex is not binary
					Binary categories for elite sporting competition are appropriate
				Information and knowledge	Information on intersex variations/androgens is lacking
			Solution	Fair play and intersex athletes	Use an athletic gender
Vilain and Martinez-Patiño [94]	2019	USA	Perspective	Information and knowledge	Information on intersex variations/androgens is lacking
				Fair play and intersex athletes	The World Athletics’ 2018 Eligibility Rules are ethically debatable
Mahomed and Dhai [95]	2019	ZA	Perspective	Fair play and intersex athletes	The regulations on hyperandrogenism from 2011 are ethically debatable
					The World Athletics’ 2018 Eligibility Rules are ethically debatable
					Excluding intersex athletes from elite sporting competition is unfair
					High androgen levels are one of many athletic advantages
Posbergh [96]	2019	USA	Perspective	The sex construct	Sex is not binary
				Fair play and intersex athletes	The World Athletics’ 2018 Eligibility Rules are ethically debatable
Wells [97]	2019	USA	Perspective	Fair play and intersex athletes	The regulations on hyperandrogenism from 2011 are ethically debatable
					A new rule for intersex athletes in elite sports is necessary
			Solution	Fair play and intersex athletes	Abolish the 2011 regulations on hyperandrogenism
					Use self-identification as a criterion to separate sporting competition
Carpenter [98]	2020	AU	Perspective	Fair play and intersex athletes	The World Athletics’ 2018 Eligibility Rules are ethically debatable
Holzer [99]	2020	CH	Perspective	The Sex Construct	Sex is not binary
Martínková [100]	2020	CZ	Solution	Fair play and intersex athletes	Set new criteria to separate sporting competition
					Use a third category to separate sporting competition
Pereira-García et al. [101]	2020	ES	Perspective	Fair play and intersex athletes	High androgen levels are one of many advantages
Schneider [102]	2020	GB	Solution	Fair play and intersex athletes	Include women in the debate
Kavoura and Kokkonen [103]	2020	FI	Solution	Fair play and intersex athletes	Set new criteria to separate sporting competition
				Information and knowledge	Educate about sex diversity
Hirschberg et al. [104]	2020	SE	Perspective	Information and knowledge	High androgen levels provide an athletic advantage
Astobiza [105]	2020	ES	Perspective	Fair play and intersex athletes	The World Athletics’ 2018 Eligibility Rules are ethically debatable
					High androgen levels are one of many athletic advantages
Brömdal [106]	2020	AU	Perspective	Fair play and intersex athletes	The World Athletics’ 2018 Eligibility Rules are ethically debatable
Loland [107]	2020	NO	Perspective	The Sex Construct	Sex is not binary
				Fair play and intersex athletes	The World Athletics’ 2018 Eligibility Rules are ethically debatable
			Solution	Fair play and intersex athletes	Set new criteria to separate sporting competition
					Use categories based on weight and height to separate sporting competition
Gollnast et al. [108]	2021	DE	Perspective	Information and knowledge	Information on intersex variations/androgens is lacking
Hamilton et al. [109]	2021	GB	Perspective	Information and knowledge	High androgen levels provide an athletic advantage
			Solution	The sex construct	Do not abolish sex segregation in sporting competition
				Fair play and intersex athletes	Intersex athletes should limit androgens to compete in the female category
					Do not use self-identification as a criterion to separate sporting competition
Krane and Waldron [110]	2021	USA	Perspective	Fair play and intersex athletes	The World Athletics’ 2018 Eligibility Rules are ethically debatable
Winkler and Gilleri [111]	2021	FR	Perspective	Fair play and intersex athletes	High androgen levels are one of many athletic advantages
					The World Athletics’ 2018 Eligibility Rules are ethically debatable
				Information and knowledge	Information on intersex variations/androgens is lacking
			Solution	Information and knowledge	Include other specialists to find a solution
Schultz [112]	2021	USA	Perspective	Fair play and intersex athletes	The World Athletics’ 2018 Eligibility Rules are ethically debatable
Mohapatra [113]	2021	IN	Perspective	Fair play and intersex athletes	The World Athletics’ 2018 Eligibility Rules are ethically debatable
Hamilton et al. [114]	2021	GB	Perspective	Information and knowledge	Information on intersex variations/androgens is lacking
			Solution	Fair play and intersex athletes	Do not use a third category to separate sporting competition
Camporesi and Hämäläinen [115]	2021	GB	Perspective	Fair play and intersex athletes	High androgen levels are one of many athletic advantages
Moyer [116]	2021	USA	Perspective	Fair play and intersex athletes	The World Athletics’ 2018 Eligibility Rules are ethically debatable
Richter-Unruh [117]	2021	DE	Perspective	Information and knowledge	High androgen levels provide an athletic advantage
					Information on intersex variations/androgens is lacking
				Fair play and intersex athletes	High androgen levels are one of many athletic advantages
			Solution	Fair play and intersex athletes	The decision on a participation permission should be conducted for each case individually
Vann [118]	2022	USA	Perspective	Fair play and intersex athletes	A new rule for intersex athletes in elite sports is necessary
					High androgen levels are one of many athletic advantages
				Information and knowledge	High androgen levels provide an athletic advantage
Shinohara [119]	2022	CH	Perspective	Fair play and intersex athletes	The World Athletics’ 2018 Eligibility Rules are ethically debatable
Chanda and Saha [120]	2022	IN	Perspective	Fair play and intersex athletes	The World Athletics’ 2018 Eligibility Rules are ethically debatable
Krane et al. [121]	2022	USA	Perspective	Fair play and intersex athletes	The World Athletics’ 2018 Eligibility Rules are ethically debatable
					High androgen levels are one of many athletic advantages
				Information and knowledge	Information on intersex variations/androgens is lacking
				Sex verification	Sex verification can cause great harm

### Substantive Analysis

A comprehensive outline of the results is presented in the following paragraphs, using the two categories: (1) perspectives and (2) proposals for solutions. Each category contains statements from the articles for each of the four themes: (1) the sex construct, (2) sex verification in elite sports, (3) fair play and intersex athletes, and (4) information and knowledge. A summary of these results is also provided in Table 2.

**Table 2:** Summary of the data synthesis

Topic	Theme	Statement	References	Distilled values
Perspectives	The sex construct	Sex is not binary	[5, 26, 32, 46, 58, 78, 93, 107, 83, 49, 67, 66, 50, 47, 57, 73, 74, 76, 80, 89, 96, 99]	Truth-finding Equality Acceptance of sex diversity
		Sex segregation in sporting competition is unjust	[58]	Social Justice Equality
		Binary categories for elite sporting competition are appropriate	[4, 93, 91]	Equity Competition Fairness
	Sex verification in elite sports	Sex verification is ethically indefensible	[6, 28, 44–48, 44, 47, 76]	Social justice Privacy
		Sex verification can cause great harm	[56, 59, 121]	Wel-lbeing Privacy Social Justice
		The athlete’s privacy must be ensured	[25, 28, 56, 52, 61, 51, 54, 88, 72]	Privacy
		Privacy is a sacrifice when competing in elite sports	[62]	Competition Fairness Dedication Compliancy
	Fair play and intersex athletes	The regulations on hyperandrogenism from 2011 are ethically debatable	[5, 7, 23, 70, 85, 71, 70–75, 75, 77, 95]	Social justice Equality
		The World Athletics’ 2018 Eligibility Rules are ethically debatable	[92, 94–96, 96, 105–107, 110–113, 116, 119–121]	Social justice Equality
		The World Athletics’ 2018 Eligibility Rules are currently the best method	[4]	Competition Fairness Equity
		Excluding intersex athletes from elite sporting competition is unfair	[58, 49, 79, 95]	Social Justice Inclusiveness Equality Recognition
		A new rule for intersex athletes in elite sports is necessary	[87, 97, 118, 122]	Recognition Social Justice Inclusiveness
		Diversity is what makes sports interesting	[78, 85]	Acceptance of sex diversity
		High androgen levels are one of many athletic advantages	[23, 78, 51, 85, 60, 83, 68, 73, 95, 101, 105, 111, 115, 117, 118, 121]	Acceptance of sex diversity Equality Recognition
		High androgen levels do not always provide an athletic advantage	[6, 28, 32, 45, 47–49, 61, 51, 48, 71, 49, 47, 63]	Education Evidence-based practice Truth-finding
		High androgen levels provide an athletic advantage	[25, 104, 109, 117, 118]	Evidence-based practice Competition Fairness
	Information and knowledge	Information on intersex variations/androgens is lacking	[4, 23, 93, 91, 85, 92, 94, 60, 53, 55, 69, 88, 71, 91–94, 108, 111, 114, 117, 121]	Evidence-based practice Truth-finding
		Information on the treatment set by IOC/World Athletics in 2011 is lacking	[71, 64]	Evidence-based practice Transparency Wel-lbeing
Proposals for solutions	The sex construct	Abolish sex segregation in sporting competition	[26, 28, 58, 52, 79]	Equality Inclusiveness
		Do not abolish sex segregation in sporting competition	[51, 109]	Equity Competition Fairness
	Sex verification	Abolish sex verification	[45, 59, 54, 67]	Social justice Privacy Equality
		Set a clear policy on sex verification	[28, 56, 88]	Transparency Equality
	Fair play and intersex athletes	Abolish the 2011 regulations on hyperandrogenism	[70, 71, 64, 97, 77]	Social Justice Inclusiveness
		Use a third category to separate sporting competition	[100]	Social Justice Competition Fairness
		Do not use a third category to separate sporting competition	[26, 91, 85, 49, 68, 66, 114]	Inclusiveness Social Justice
		Intersex athletes should limit androgens to compete in the female category	[25, 58, 61, 51, 109]	Equity Competition Fairness
		Use an athletic gender to separate sporting competition	[4, 93]	Social Justice Competition Fairness
		Do not use testosterone as a criterion to separate sporting competition	[5, 91]	Acceptance of sex diversity
		Set new criteria to separate sporting competition	[26, 107, 100, 72, 67, 63]	Evidence-based practice Acceptance of sex diversity Inclusiveness Competition Fairness
		Include women in the debate	[72, 102]	Inclusiveness Equality
		Treat all advantages in sporting competition equally	[67]	Equality
		Use categories based on weight and height to separate sporting competition	[107, 90]	Competition Fairness
		Do not use biological parameters as criteria for separating sporting competition	[5]	Equity Inclusiveness
		Use self-identification as a criterion to separate sporting competition	[78, 84, 97, 50]	Autonomy
		Do not use self-identification as a criterion to separate sporting competition	[25, 83, 109]	Competition Fairness
		The decision on a participation permission should be conducted for each case individually	[117]	Social Justice Equity
	Information and knowledge	Educate about sex diversity	[44, 103]	Education Recognition Evidence-based practice
		Include other specialists to find a solution	[28, 56, 61, 60, 49, 82, 111]	Evidence-based practice Recognition

#### Perspectives

##### The Sex Construct

*Sex is not binary:* The authors of 22 articles indicate a nonexistence of binary sex categories in nature (Table 2). For example, Wackwitz [46] points out that individuals who do not fit into one of the two socially ascribed categories are commonly forced into one and made to conform, physically and psychologically. She criticizes how these individuals are not considered to have their own biological identities, and how they are instead described as a mixture of male and female parts.

*“As a binary system—a constructed and socially imposed binary system—the struggle is to preserve the integrity of that system as it is designed against the reality of life as it is lived.”* [46]

Foddy and Savulescu [58] refer to Caster Semenya, stating that her case has raised the following problem: whether someone is male or female does not always have a binary answer. Buzuvis [78] claims that a separation of humans into male and female categories using hormones or other physiological characteristics as parameters is not possible.

*“Human beings do not fall neatly into male and female categories that can be objectively and conclusively determined by hormones or any other physiological characteristic.” *[78]

Harper et al. [93] contend that every human gets sorted into a male or female bin after birth. Therefore, they claim that deciding who is and is not allowed to compete in the female category is one of the most complex and emotional topics in elite sports in recent times.

*Sex segregation in sporting competition is unjust:* Foddy and Savulescu [58] take the view that sex segregation in sports should be perceived as an inconsistent and unfair policy, due to a nonexistent binary gender.

*“But once we recognize that gender is not a binary quantity, sex segregation in sports must be seen as an inconsistent and unjust policy, no matter what stance we take on the goals of sport or on the regulation of doping.” *[58]

*Binary categories for elite sporting competition are appropriate:* Jakob [91], Harper et al. [4], and Harper et al. [93] assert that binary categories in elite sports are appropriate. More specifically, Harper et al. [93] state that separating all athletes into male and female categories is the only way to uphold the Olympic Charter and ensure meaningful sporting competition for women athletes.

*“In conclusion, to uphold the Olympic Charter and ensure meaningful sporting competition, it is necessary to use an evolving evidence-based scientific method to separate athletes into male and female categories.” *[93]

##### Sex Verification in Elite Sports

*Sex verification is ethically indefensible:* The authors of 11 articles claim that the practice of sex verification is ethically indefensible (Table 2). For example, Dickinson et al. [45] state that the sorting of women based on only laboratory results was discriminatory and resulted in emotional trauma.

*“Problems include invalid screening tests, failure to understand the problems of intersex, the discriminatory singling out of women based only on laboratory results, and the stigmatization and emotional trauma experienced by individuals screened positive.” *[45]

Adair [28] mentions discrimination, entailed by sex verification tests, by referring to what happened to the athlete Caster Semenya.

*Sex verification can cause great harm:* The authors of three articles claim that sex verification can cause great harm. Wonkam et al. [56] and Wiesemann [59] refer to individuals who, prior to verification, are unaware of their intersex status. More specifically, Wonkam et al. [56] mention the psychological harm that unknowing women might encounter.

*“Gender verification has potential for causing great psychological harm to women who may unknowingly have a Disorder of Sexual Development […]” *[56]

Wiesemann [59] adds that there is no possibility of going back to a state of not knowing about one’s intersex status.

*The athlete’s privacy must be ensured:* The authors of nine articles take the view that privacy must be ensured (Table 2). Merck [52] claims that no one, except a doctor, needs to know a person’s sex. For all others, a person’s gender should be decisive.

*“[…] a person’s sex is something only a doctor needs to know to provide adequate health care. All we need to know as friends, colleagues, family members, fans, etc. is the gender identity of the person.” *[52]

Ballantyne et al. [61] share this attitude, stating that the causes and consequences of a high testosterone level can be dealt with in private. Wonkam et al. [56], Hercher [51], and Adair [28] highly criticize the managing of privacy in Caster Semenya’s case. In more detail, Wonkam et al. [56] assert that the policy must protect the rights and privacy of athletes. Saleem [54] refers to safeguards resembling the confidentiality rules of international human rights declarations, stating that they are needed to protect the anonymity and autonomy of professional athletes.

*“Finally, the gender verification rule should implement safeguards that protect the anonymity and autonomy of professional athletes. Safeguards that resemble confidentiality rules of international human rights declarations will not only protect athletes from public humiliation, but also regain trust within the sporting government” *[54]

*Privacy is a sacrifice when competing in elite sports:* Pierson [62] takes the view that the only way to retain an athlete's privacy is to stop competing. Moreover, he claims that there exists no such “right to compete.” Thus, if an athlete decides to enjoy the privilege of competing at an elite level, he or she must sacrifice certain rights.

*“No such “right to compete” exists. In stark contrast, once one has achieved an elite level of performance, it is explicitly stated that it is a privilege to compete, and in order to enjoy such a privilege; the sacrifice of certain rights is required. One of these sacrifices is to submit to various verifications and tests as determined by any number of governing bodies. This includes the invasion of privacy in the form of randomized drug testing […], age-verification processes and, in certain instances gender-verification procedures.” *[62]

##### Fair Play and Intersex Athletes

*The regulations on hyperandrogenism from 2011 are ethically debatable:* The authors of 15 articles claim that the regulations on hyperandrogenism set by IOC/World Athletics in 2011 (see Introduction) are ethically debatable (Table 2). Dabholkar [70] takes the view that asking intersex athletes to undergo hormonal treatment before participating in international competitions is extremely discriminatory. Ingthorsson [85] states that requesting someone to change their natural being is morally disputable.

*“However, I agree with Tamburrini that it is morally questionable to offer anyone the option of undergoing chemical treatment to “correct” how they are by nature.” *[85]

*The World Athletics’ 2018 Eligibility Rules are ethically debatable:* The authors of 16 articles claim that the options forced by the new Eligibility Regulations on intersex elite athletes, set by World Athletics in 2018, are ethically debatable (Table 2). Karkazis and Carpenter [92] assert that the new rules violate the athlete’s dignity, threaten their privacy, and spread both suspicion and judgment regarding their sex and gender identities. Moreover, they indicate that the rules would be enforced through humiliation, stigmatization and fear.

*“The new World Athletics regulations not only fail to uphold dignity, privacy, and fairness for all women athletes, they violate these principles and more generally hamper athlete participation.” *[92]

Vilain and Martinez-Patiño [94] criticize the regulation, stating that the athletes affected did not choose to have the trait. Moreover, they observed the regulations are restricted to some events; other events, such as the hammer throw, are left out, even though the study indicated a significant difference in performance between athletes with high and normal testosterone levels in such events. The aforementioned study, published by Bermon and Garnier [36], proves a significant relation of high androgen levels and athletic performance in 400 m and 800 m sprint, 400 m hurdles, pole vault and hammer throw. Loland [107] describes his support of the DSD regulations as conditional. On the one hand, he understands the need for classification, on the other hand, from an ethical perspective, he claims that the requirements are difficult to support.

*The World Athletics’ 2018 Eligibility Rules are currently the best method:* Harper et al. [4] state that the new Eligibility Rules set by World Athletics in 2018, while imperfect, are currently the best available method to fairly separate male and female athletes. They base this view on the above-mentioned study published by Bermon and Garnier [36].

“Nevertheless, the new DSD regulations are a perfectly reasonable attempt to create legislation ensuring “fair and meaningful competition’ for all women.” [4].

*Excluding intersex athletes from elite sporting competition is unfair: *The authors of four articles claim that the exclusion of intersex elite athletes is unfair (Table 2). Foddy and Savulescu [58] link this argument to Caster Semenya stating that there is no reason to exclude athletes who, like her, have not violated any rules.

*“[…] there is no justification for excluding an intersex athlete, perhaps like Caster Semenya, who has broken no rules, and whose only crime is that they are not at an extreme of the gender spectrum.” *[58]

*A new rule for intersex athletes in elite sports is necessary:* The authors of three articles demand the establishment of a new rule for intersex athletes in elite sports (Table 2). Ljungqvist [87] emphasizes the establishment of a new rule prior to the 2018 Eligibility Rules. He supports his demand by citing the rapid developments in science and society that have led to an increasing number of countries changing their regulations on legal sex, especially the recognition of a third sex.

*“Both the IOC Executive Board and the World Athletics Council have found that a rule on female hyperandrogenism is necessary. This is particularly true at a time when rapid developments in science and society are leading to an increasing number of countries liberalising their regulations on assignment [and reassignment] of legal sex, and [in particular] recognising a third sex. Sport will have to meet those challenges by putting in place adequate rules in order to protect the integrity and fairness of sport competitions for women.” *[87]

*Diversity is what makes sports interesting:* Buzuvis [78] and Ingthorsson [85] claim that diversity is what makes sport interesting. Buzuvis [78] states that the diverse distribution of physical, psychological, environmental and social characteristics is essential to sport.

*“It facilitates the myth that a level playing field is something that sport can and should construct, instead of acknowledging the reality that the diverse distribution of physical characteristics [not to mention psychological, environmental, and social ones] are essential to sport. That diversity is what makes sport outcomes unpredictable and the contest itself worthwhile.” *[78]

*High androgen levels are one of many athletic advantages:* The authors of 15 articles claim that high androgen levels are one of many athletic advantages (Table 2). Hercher [51] takes the view that there is nothing wrong with this genetic advantage, observing that genetic advantages are standard in competitive sports.

*“Taking an excess of testosterone is cheating. Producing an excess of testosterone is a genetic advantage, and there is nothing inherently wrong with that. Genetic advantages are the norm and not the exception in competitive sports.” *[51]

Schultz [60] shares this opinion stating that competitors never begin on an even playing field. Elite athletes, she claims, all possess some kind of advantage over the general population. For her, it does not make a difference whether an advantage is circumstantial, cultural, psychological, or biological. Buzuvis [78] criticizes the sport federations for not having focused on other, non-biological factors that might result in athletic advantages. Moreover, Ingthorsson [85] and Pielke [83] criticize the sole focus on androgens stating that other biological issues, such as height, can also provide a competitive advantage.

*High androgen levels do not always provide an athletic advantage:* The authors of 14 articles claim that high androgen levels do not always provide an athletic advantage (Table 2). Notably, most of these articles were published prior to the regulations on hyperandrogenism in 2011, which then applied solely to athletes with androgen sensitivity. Reeser [48] indicates that the presence of the Y chromosome and circulating testosterone do not necessarily result in a competitive advantage. With this statement, he refers to individuals with androgen insensitivity. Hercher [51] states that neither intersex variation, congenital adrenal hyperplasia (CAH) or androgen insensitivity syndrome (AIS) has any bearing on an individual’s ability to compete. Dickinson et al. [45] share this view and explicitly refer to the intersex variation 5-alpha reductase deficiency, partial or complete androgen insensitivity syndrome and chromosomal mosaicism.

*“Individuals with sex-related genetic abnormalities raised as females have no unfair physical advantage and should not be excluded or stigmatized, including those with 5-alpha-steroid–reductase deficiency, partial or complete androgen insensitivity, and chromosomal mosaicism.” *[45]

Amy-Chinn [32] references Caster Semenya stating that even if Semenya does have an increased level of testosterone, any intersex variation she might have is unlikely to provide an unfair advantage.

*High androgen levels provide an athletic advantage:* The authors of three articles claim that testosterone is performance-enhancing. Auchus [25] supports this statement by citing Bhasin et al. [123] landmark study, which proved that the use of androgen T-enanthate increased muscle strength and size in young healthy male athletes.

*“The evidence that T is a performance-enhancing substance is irrefutable. In a landmark study, for example, Bhasin et al. demonstrate that with or without exercise, use of the androgen T-enanthate at 600 mg/week profoundly increases muscle strength and size over a placebo in young healthy male athletes.” *[25]

Hirschberg et al. [104] present their own findings on the effects of moderately increased testosterone concentration on physical performance in young women. The findings indicate that testosterone affects aerobic performance by promoting a leaner body composition with increased muscle mass.

##### Information/Knowledge

*Information on intersex variations /androgens is lacking:* The authors of 22 articles highlight the lack of information on androgens and intersex variations (Table 2). Glazer [23] claims that it is unclear whether high androgen levels result in a competitive advantage. Nerva [53] describes a lack of data on the relationship between resting testosterone levels and neuromuscular performance. Tucker and Collins [55] indicate that the most important missing component is evidence of a possible advantage in performance for elite athletes with DSD and the extent of this advantage.

*“Finally, the most important missing component of this debate is the sound scientific evidence to determine [a] whether a performance advantage exists and [b] how large it may be.” *[55]

Gandert et al. [69] state that any athletic advantages naturally possessed by an intersex competitor might be of a permissible kind. Ingthorsson [85] describes the assumption that intersex women are physically superior to other athletes as entirely hypothetical. Fields [88] demands more research on intersex athletes.

*Information on the treatment set by IOC/World Athletics in 2011 is lacking:* As a reminder, the regulations on female hyperandrogenism set in 2011 required that elite athletes with high androgen levels wanting to compete in the female category undergo treatment to lower their testosterone level to less than 10 nmol/L. Ferguson-Smith and Bavington [71] and Viloria and Martinez-Patino [64] consider the information on this treatment to be too fragmentary; they fear the health issues that might result from this treatment.

*“In addition, there is no evidence that the treatments athletes who are deemed ineligible will be required to undergo in order to compete will not be harmful to their health.” *[64]

#### Proposals for Solutions

##### The Sex Construct

*Abolish sex segregation in sporting competition:* The authors of five articles consider the abolishment of sex segregation in sporting competition. Foddy and Savulescu [58] discuss the objectives of athletic sport. They state that if athletics aims to identify an athlete’s natural potential, sex segregation should be abolished. Adair [28] proposes that governing bodies of athletic competitions should avoid the issue around sex segregation all together, from an early age through to elite sports. Moreover, she suggests that the integration of intersex athletes must already occur in physical education prior to high school.

*“In order to avoid the legal quandary presented by intersex athletes and the inevitable trauma inflicted on the excluded athlete, the governing bodies of athletic competitions should seek to avoid the issue all together, from an early age through elite competition. By integrating all genders in physical education activities prior to high school, the issue of intersexual inclusion becomes irrelevant.” *[28]

*Do not abolish sex segregation for sporting competition:* Hercher [51] criticizes the idea of abolishing sex segregation. She states that having a dividing line between the sexes is a question of fairness.

*“[…] then there has to be a dividing line. This is a question of fairness on the playing field and not a question of dictating appropriate limits for gender identity.” *[51]

Hamilton et al. [109] share this view. They assess that a separation on the basis of biological sex is necessary due to some benefitting from the effects of testosterone, whereas others do not.

##### Sex Verification in Elite Sports

*Abolish sex verification:* The authors of four articles propose abolishing sex verification (Table 2). Wiesemann [59] specifies that sex verification should be abolished as soon as possible and only be reconsidered if the consequences are less harmful.

*“Therefore, genetic sex determination in sports should be abolished as soon as possible. Testing may only be reconsidered if the harm inflicted upon individual persons is substantially reduced.” *[59]

Dickinson et al. [45] explicitly refer to sex verification tests based on chromosomes, since no male imposters were detected at international events that used X chromatin analysis or SRY testing, the sequencing of the sex-determining region Y.

*Set a clear policy on sex verification: *Adair [28], Fields [88], and Wonkam et al. [56] demand a clear policy on sex verification. Fields [88] indicates that an appropriate procedure and clear guidelines are needed to solve the topic of intersex athletes competing in the female category. By referring to Caster Semenya and similarly situated intersex athletes, Adair [28] demands a clear policy on the sex verification process, to prevent intersex exclusion and discrimination.

*“By implementing responsible gender policies to prevent intersex exclusion and rectifying discrimination through the judicial system, Caster Semenya and similarly situated intersex athletes will remain in their rightful place: on the track, rather than in the courtroom.” *[28]

##### Fair Play and Intersex Athletes

*Abolish the 2011 regulations on hyperandrogenism:* The authors of five articles call for the abandonment of the policies set by the IOC/World Athletics in 2011 (Table 2). For example, Viloria and Martinez-Patino [64] demand the abandonment of these policies before more female athletes are harmed. Moreover, they propose letting all athletes who have grown up and lived as females compete in the female category without any sanctions against them.

*“[…] and that all athletes who have grown up and continue to live as female be eligible to compete as such without having sanctions imposed against them.” *[64]

*Use a third category to separate sporting competition*: To separate sporting competition, Martínková [100] considers the use of a third, unisex category.

*“[…] or leave the existing sport as it is and add a new unisex category (having three sex categories: male, female and unisex; or four categories: male, female, mixed and unisex). However, the new unisex category will differ from the sex category, since it will rather be a modified version of the sport.” *[100]

*Do not use a third category to separate sporting competition:* The authors of seven articles reject the idea of using a third category to separate sporting competition (Table 2). Camporesi and Maugeri [49] discuss the option of a third category that would encompass individuals with any variations related to sexual development, concluding that this would simply be impractical and discriminatory.

*“Another option might be devised by anyone wishing to preserve strict sexual boundaries namely, to create a brand new category for any disorder or syndrome related to sex! We suspect this will not happen, because it would be both impractical and discriminatory.” *[49]

Ingthorsson [85] asserts that a separate class is not possible due to the small population of intersex athletes wanting to compete in the female category. Sánchez et al. [68] reject a third category because it could further stigmatize athletes who are not even proven to possess an athletic advantage.

*Intersex athletes should limit androgens to compete in the female category:* The authors of five articles suggest a limitation of androgens in order to compete in the female category in elite sports (Table 2). Already before the IOC and World Athletics set their regulations on hyperandrogenism in 2011, Hercher [51] had proposed a similar approach. In her article, she refers to the existing guidelines for athletes after male-to-female sex reassignment, which permit the participation after gonadectomy and two years of hormone therapy. She suggests intersex athletes with functioning testes and no androgen insensitivity could be required to take similar measures if they intend to compete in the female category. Auchus [25] also addresses the regulations set by the IOC and World Athletics in 2011 and proposes lowering the 10 nmol/L cutoff to 3 nmol/L. He claims that this limit is closer to the upper limit of the female range. Responding to the United Nations Human Rights Council’s Report on Race and Gender Discrimination in Sport, Hamilton et al. [109] express the opinion, that circulating testosterone levels of athletes with DSD that are far above the normal female level should be lowered. They justify their assessment with the desire to maintain fairness while enabling inclusion.

*“To have meaningful competition, our current opinion is that the much higher than the normal female range in circulating testosterone levels in DSD needs to be mitigated *[17, 20]*. This action is intended to achieve a balance of fairness and safety while permitting inclusion, as reducing testosterone will reduce or eliminate the advantages conferred by androgens during puberty and development *[21]*. These measures are consistent with the idea that elite female competition forms a “protected category” with an entry that must be restricted by objective eligibility criteria.”*[109]

*Use an athletic gender to separate sporting competition: *Harper et al. [93] and Harper et al. [4] suggest the use of an athletic gender for elite sports competitions. The idea behind this proposal is to use serum testosterone levels to divide all athletes into male and female categories. Harper et al. [93] support this recommendation by stating that legal gender cannot be the sole criterion in determining sporting categories. Moreover, they claim that viewing the separation of athletes into male and female categories as an athletic gender helps solve the complex topics around sex, gender, and sport.

*“It is helpful to view the separation of athletes into male and female categories as the determination of an athletic gender. If the idea of an athletic gender is adapted, the increased use of this concept will result in clearer sporting policies and a reduction in the discord between various factions in the very complex world of sex, gender, and sport.” *[93]

*Do not use testosterone as a criterion to separate sporting competition:* Newbould [5] and Jakob [91] reject the idea of forming two groups according to testosterone levels. Specifically, Jakob [91] claims that the idea is inexpedient. Newbould [5] fears that the introduction of a testosterone rule would lead to the medicalization of sport.

*“However, such a method is unlikely to be of any practical use. The advantage conferred by hyperandrogenism is complex and a single or a few measurements of testosterone in isolation may carry very little significance. Therefore, this solution is unlikely to offer any advantage and would have the effect of increasing the medicalisation of sport to a degree that many might consider unacceptable.” *[5]

*Set new criteria to separate sporting competition:* The authors of seven articles demand new criteria to classify elite sporting competition. Teetzel [72] states that new criteria on the basis of science and in line with ethical research are needed.

*“[…] attempts to design new criteria for inclusion, based on science and in line with the highest standards of ethical research, are needed.” *[72]

Cooky and Dworkin [67] indicate that sex is not the only possible way to organize competition categories in sports. They add that sports should be sorted according to the abilities of the athletes, instead of stereotypical attributes. Martínková [100] makes a similar proposal, rejecting the binary division of sports. Instead, she suggests modifying certain criteria to enable one unisex sport. Wiederkehr [66] refers to disabled sports suggesting it could be an orientation for elite sports to focus on the individual physical features instead of male or female sex.

*Include women in the debate:* Teetzel [72] and Schneider [102] demand the inclusion of female athletes in the debate. Teetzel [72] asserts that female athletes must be heard even though their views might be perceived as politically incorrect.

*“As debate continues, we must ensure that women athletes’ voices are heard on this issue, even if the perspectives expressed may be viewed as politically incorrect.” *[72]

Schneider [102] claims that the community of female athletes should have the most prominent voice in the discussion and that men’s voices, although they should be heard, should not be the deciding factor.

*Treat all advantages for sporting competition equally:* Cooky and Dworkin [67] suggest that, if a level playing field is desired, all genetic and pacing advantages should be treated equally.

*“If a level playing field is desired [or even possible], then we posit sport-governing bodies and sport organizations should treat all genetic advantages and all pacing advantages equally.” *[67]

*Use categories based on weight or height to separate sporting competition:* The authors of two articles suggest the use of weight or height to separate certain sports. Loland [107] claims that there should be no sex classification in those disciplines, in which biological sex does not have a significant impact. Because they use weight classes in weight lifting and combat sports, he proposes that there could be height classes in sports such as high jumping, basketball or volleyball. Virgilii [90] suggests the decoupling of sports categories from biological sex, stating that an individual’s biological sex does not necessarily match their gender, and that neither biological sex nor gender are binary. For boxing, she proposes the use of categories based on weight and height instead.

*“The proposal is to form the sports categories according to different parameters from those of biological sex and gender, in this particular case based on weight and height.” *[90]

*Do not use biological parameters as criteria for separating sporting competition:* Newbould [5] criticizes the idea of using biological parameters, such as height or weight. According to her, it would be difficult and does not guarantee women’s participation.

*“Any biological parameter or combination of parameters could be used, such as height or weight or a combination of them. It might be possible to devise a system of having multiple categories, based on physical abilities and parameters, in a similar way to the system used in the Paralympics, where physical impairment is classed into one of eight groups. However, the argument against this system is that it would be complex to administer and would not guarantee women’s participation.” *[5]

*Use self-identification as a criterion to separate sporting competition:* The authors of four articles suggest allowing individuals to participate according to self-identification. Buzuvis [78] restricts this option to non-transgender women. Katz and Luckinbill [84] add the idea of more available options despite the current male and female categorization.

*“In athletic competitions, individual competitors should be allowed to participate as the gender that most closely approximates how each participant identifies. Currently the options are male and female, but there may be more options available as gender fluidity evolves.” *[84]

Wells [97] and Dreger [50] claim that intersex individuals who have lived their entire lives as women should be allowed to compete in the female category.

*Do not use self-identification as a criterion to separate sporting competition:* The authors of three articles disagree with the proposal of using self-identification as a criterion. Pielke [83] states that this approach would increase the chances of fraud. Auchus [25] also rejects the proposal because he fears athletes would go to extreme measures to win medals.

*“Let anybody enter the women’s competition. This option might sound absurd. Basing participation on gender, not sex, would open the floodgates for the many male athletes who will go to extreme measures to win medals.” *[25]

*The decision on a participation permission should be conducted for each case individually: *Because DSD variations are diverse, Richter-Unruh [124] proposes that the decision on whether an athlete with DSD is allowed to start, ought to be based on the individual case. Moreover, it should be made together with the athlete, by an independent and interdisciplinary commission.

##### Information and Knowledge

*Educate about sex diversity:* Elsas et al. [44] and Kavoura and Kokkonen [103] propose the education of the actors involved. Specifically, Elsas et al. [44] suggest continuously educating athletes, sport governing bodies, medical delegates, and team physicians on the biological complexity of sex.

*“We endorse the continued education of athletes, sports governors, medical delegates, and team physicians concerning the biological complexities of sex differentiation.” *[44]

Kavoura and Kokkonen [103] propose the education of all actors involved, not explicitly about intersex individuals/biological variations but, rather, about the avoidance of reinforcing heteronormativity.

*Include other specialists to find a solution:* To find a suitable solution, the authors of seven articles demand the inclusion of other specialists. Schultz [60] states that insights from biomechanics, philosophy, psychology, physiology, motor control, history, and sociology are needed for a more holistic understanding of sex and athletic advantages. Ballantyne et al. [61] strive for a discussion involving experts from the fields of biology, medicine, genetics, psychology, sport, and ethics.

*“Rather, we aim to contribute to an open discussion involving experts from the fields of biology, medicine, genetics, psychology, sports and ethics, to accomplish a procedure which would respect the authenticity of an adult individual’s sex and gender identity.” *[61]

Lovett [82] suggests asking transgender women for help to solve the current situation. In the explanation of his proposal, he states that they know exactly what it means to be female.

*“I find it intriguing, and in the finest sense ironic, that the best handle we may have on this begins with transgender women. If science pans out, it is likely to be these people – not the ones born in bodies that matched their inner gender identities – who may help us understand exactly what it means to be female.” *[82]

### Distilled Values

**Fig. 3:**
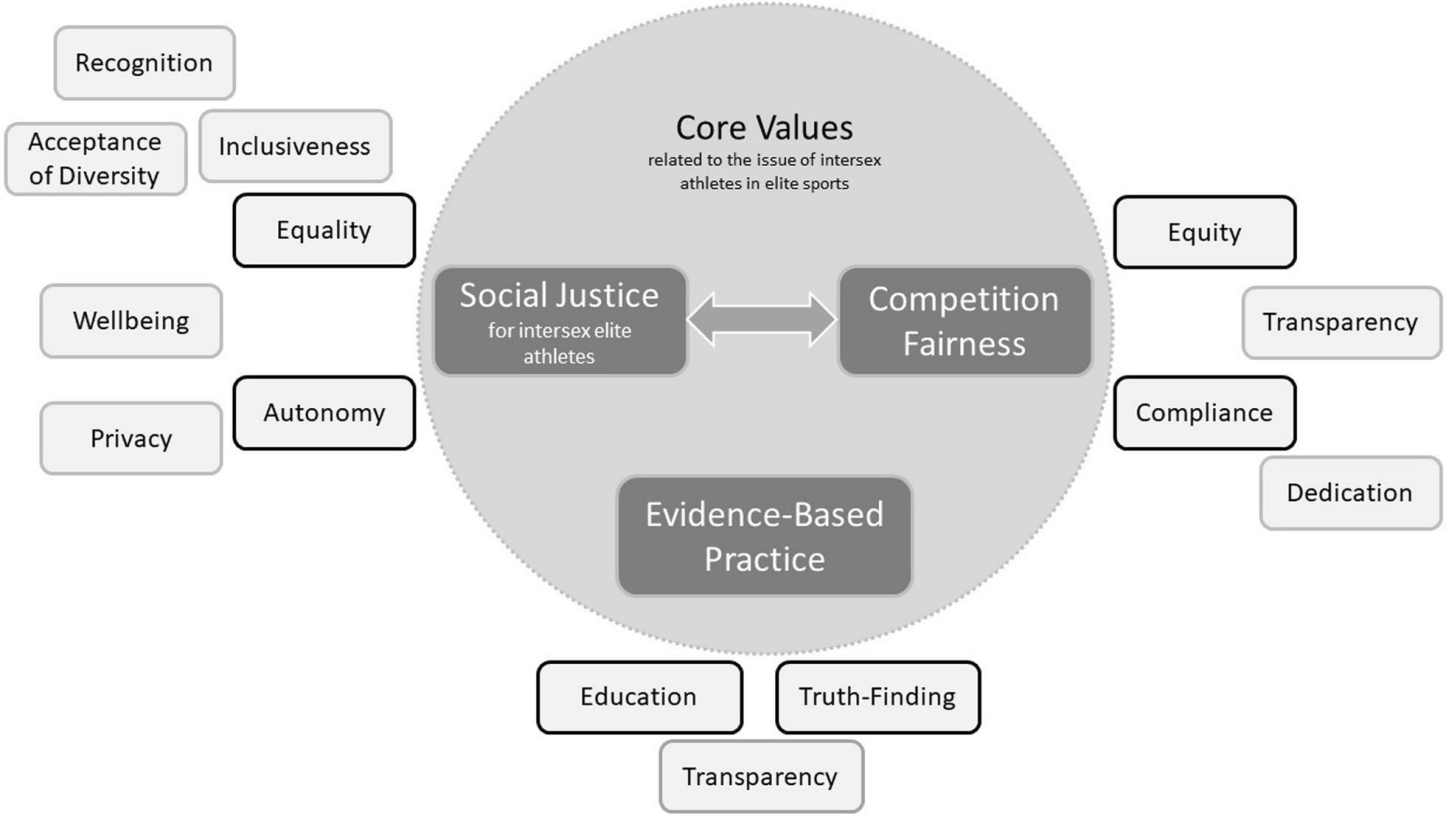
Distilled core values presented in the academic literature (January 2000 to July 2022) regarding intersex athletes in elite sport

In total, 16 different values were extracted from the authors’ statements presented in the included articles (Table 2). Axial coding revealed three so-called core values: social justice for intersex elite athletes, competition fairness, and evidence-based practice (Fig. 3). Social justice for intersex elite athletes refers to the concept of fair and just relations between an individual and society, including the values of equality and autonomy. Competition fairness is about the virtue of justice and equity for all athletes during any competition and rule adherence whereby athletes abide by the rules of competition (i.e., compliancy). The last, evidence-based practice, refers to the acquisition and the use of knowledge based on scientific studies. These three are proposed to form the essential values on which the statements within the included articles are built. The other distilled values form the outer rim representing the core values’ different aspects. In addition, it is worth mentioning that the statements within the included articles disclose an interaction, or even a conflict, between the core values of social justice for intersex elite athletes and competition fairness, especially when seen from different perspectives. For example, excluding athletes with intersex variations and relatively high testosterone levels from competition might be considered a means to increase competition fairness. Still, it contradicts the value of inclusiveness in social justice.

## Discussion

This systematic review aimed to discover how the topic of intersex athletes within elite sports is positioned in the academic literature from January 2000 to July 2022 from a neutral perspective. Some general aspects of the results, regarding the systematic search and the data synthesis, are worth mentioning. First, a closer look at the journals’ titles extracted from the systematic search indicates different thematic emphases. Most frequent are journals with a medical emphasis, probably because the regulations on intersex athletes in elite sports primarily depend on medical issues, such as the chromosome pattern or the testosterone level. Despite this, the results also contain many articles from journals that emphasize law, ethics, or philosophy, indicating the presence of a multidisciplinary problem.

Second, as illustrated in Fig. 2, the publication rate varies widely during the period examined (January 2000 to July 2022). Different events in the interim can explain this asymmetry. For instance, the rapid increase in 2010 presumably resulted from Caster Semenya’s first public appearance, the beginning of the controversy around her femaleness/sex [32, 33]. The second rise in the rate of publications followed in 2018, the year after Bermon and Garnier [36] published their study about the relationship between high androgen levels and sporting performance in November 2017. The study was given particular attention because the previous regulations on hyperandrogenism were suspended in 2015 due to insufficient data [35]. World Athletics directly reacted to the study’s results by releasing new regulations in April 2018 [38]. The highest increase in 2021 can be explained by the challenge Caster Semenya filed against the CAS, which was suspended in May 2019, and her most recent appeal to the ECHR in February 2021. Additionally, the generally increasing publication rate between 2000 and 2022 can be interpreted as the growing interest in intersex athletes in elite sports.

Finally, it was found that the article’s authors stem from sixteen different countries. At first sight, this finding suggests a global scope. A closer look shows an uneven distribution as 37 of 87 corresponding authors were affiliated in the USA and another 16 in Great Britain. Presumably, this likely resulted from the inclusion criteria of texts being in English or German. This also explains Germany being the third most common country in this study.

The results of the substantive analysis provide an overview of the statements found in the included articles and reveal different perspectives toward intersex elite athletes in the last decades and multiple proposals for solutions, which sometimes appear contradictory. First, it is notable that both perspectives and proposals for solutions show great agreement throughout the timeline. This is most likely due to the decisive events, such as the controversy surrounding Caster Semenya or the different regulations set by the IOC and World Athletics. Of course, changes throughout the time period can be identified, since the articles focus on the different topics that appeared to be relevant: sex verification tests, the regulations from 2011 and 2018, as well as Semenya’s appeal against the CAS and the ECHR. Also, the increasing scientific knowledge concerning intersex individuals and the importance of androgen levels strongly impacted the articles.

However, a closer look at the individual themes emphasizes the topic’s complexity. The conclusion of the perspectives points out the authors’ criticism. It primarily concerns the categorization in elite sports, especially its underlying binary sex concept. As a result of the binary separation in elite sports, the participation of intersex athletes is associated with complications and temporarily led to sex verification tests for the female category and regulations requiring exclusion or hormone treatment for athletes being intersex and/or hyperandrogenic [18]. Several authors criticized the IOC and World Athletics by claiming their regulations from 2011 and 2018 are ethically debatable. Moreover, the information about intersex variations and androgens combined with sporting performance is declared insufficient [55, 85, 92]. As for the proposals for solutions, the authors indirectly react to each other’s proposals by promoting, modifying, or rejecting them. They include discussions on how to enable the continuing binary division of all athletes or on how to categorize elite sports instead. Still, none of these proposals appear to satisfy all requirements. The lack of research, which is mentioned in several articles throughout the time period, makes it even more difficult to establish a sound solution. Therefore, several authors advocate for additional scientific research and the inclusion of specialists from other fields in the discussion. These findings are underlined by the distilled values, which vary depending on the ideas expressed.

The qualitative approach for data analysis revealed three core values on which the statements are built: social justice for intersex elite athletes, competition fairness, and evidence-based practice. These cornerstones show that the articles do include not only more scientific related values (i.e., evidence-based practice) but also cultural values (i.e., social justice) and values related to the specific context of sports (i.e., competition fairness). Most striking is the tension between competition fairness, by creating a level playing field, and the striving for social justice for intersex elite athletes, fairness, and integrity. Interestingly, equity and competition fairness are often connected, which can be explained by the intention to create a level playing field by producing equity for all athletes. Subsequently, hormone treatment or the exclusion from competition serves as an instrument to create the intended equity. On the other hand, social justice for intersex elite athletes is often connected with equality. In contrast to equity, equal treatment of all athletes in the female category would result in social justice for intersex individuals. It becomes evident that sports federations currently find themselves amidst the tension between social justice for intersex elite athletes and competition fairness, needing to decide in favor of one. Can there be a sound solution that enables the inclusion of all athletes and a level playing field simultaneously, as is desired by the Fundamental Principles of Olympism?

This raises the question of what the exact role of science within the topic of intersex athletes in elite sports should be. Science can deliver evidence for both sides of the coin (i.e., social justice and competition fairness). According to Zohoor [125], most scientists believe that one basic characteristic of science is it deals with facts, not values. One major reason for this is the assumption of values not being objective, whereas science is. But, in practice, values can be objective if they underlie accepted principles, while science comprises cultural values [125]. Additionally, ethical concern and social values become involved in the use of results of research, as well as in methods or the practice of science [125]. As indicated in the Results section, the debate about intersex athletes in elite sports goes beyond simply measuring chromosome patterns and androgens. The distilled values point to the importance of an ethical discussion and the need for sound scientific data. It is a multidisciplinary problem and therefore requires further examination and assessment via a diverse multidisciplinary team (e.g., sport practice, medicine, sport science, law, social science, and humanities).

Currently, the question of how to address intersex athletes seems to be a dilemma specific to the field of sports, even though other fields also use binary categorizations. The main reason for this is the focus on physical aspects and the highly competitive environment that comes with the sport. Another noticeable constraint is the sole focus on women’s sports, whereas the male category seems uninvolved/omitted. As Krane [126] highlights, atypical male bodies (e.g., long arms, height) are celebrated, and success is explained by exceptional mechanics, mental toughness, and training. A digression to female transgender athletes in elite sports reveals a similar challenge as with intersex athletes: how to deal with the binary division and athletic advantages [4, 93]? Recent results by Harper et al. [127] and Hilton and Lundberg [128] indicate an insufficient suppression of male performance advantages in transgender athletes after 12 and 36 months of hormone therapy. They raise the question of whether this also concerns intersex athletes.

Four potential limitations should be acknowledged that might have influenced the results. First, generalization of the results should be made with some caution due to the exclusion of all articles that were not written in either German or English. Although English is the predominant language in science, as Drubin and Kellogg [129] indicate, there are still many articles that are only accessible in other languages. Moreover, it needs to be mentioned that the statements regarding the perspective or search for a solution are only short extracts from each article and probably to some extent subject to the selectivity of the current authors. Therefore, conclusions about the individual papers should be made with caution. Third, only written statements within the published articles were included. One could argue whether this fully represents how intersex biological variation is positioned in science. A more in-depth study design (e.g., semi-structured interviews with scientists) could shine a light on this matter. Fourth, as mentioned before, a closer look at the journals extracted from the systematic search indicates that many have a medical emphasis. However, the topic is a multidisciplinary problem. Future publications in other fields could reveal different perspectives and proposals for solutions.

## Conclusions

In conclusion, the results provide an overview of the authors’ statements, reflecting how the subject of intersex athletes in elite sports has been positioned in the academic literature between January 2000 and July 2022. Moreover, they provide a better understanding of what appears to be relevant for intersex athletes in elite sports. The perspectives point out criticism, which merely concerns the binary categorization in elite sports, the regulations set by the IOC and World Athletics, and a lack of research on intersex variations. Even though most of the approaches show similarities, there are also several contradictory statements. The proposals for solutions include discussions on how to enable the binary division of all athletes and offer alternative categorizations for elite sports. The underlying values indicate the topic’s complexity. The main conflict between the approaches to creating social justice for intersex elite athletes while maintaining competition fairness is noticeable.

A closer look at these values entails a discussion on the role of science within the topic of intersex athletes in elite sports. The importance of other approaches within science and other fields than the current medical focus becomes evident. Therefore, we propose that medical science should not be considered the sole academic stakeholder on the topic of intersex athletes in elite sports. Instead, the solution-finding process needs a multidisciplinary approach including scientists and other experts/stakeholders with diverse backgrounds from, among others, medical science, ethics, social sciences, and sports practice to build a broad social consensus. Additionally, more awareness and better education on intersex variations would contribute to a better understanding of the complex topics and a respectful approach for all involved [125].

## Data Availability

The data that support the findings of this study are available from the corresponding author upon reasonable request.

## Abbreviations

DSD: Differences (or disorders) of sexual development
IAAF: International Association of Athletics Federations
IOC: International Olympic Committee
CAS: Court of Arbitration for Sport
ECHR: European Court of Human Rights
PRISMA: Preferred reporting items for systematic reviews and meta-analyses
CAH: Congenital adrenal hyperplasia
AIS: Androgen insensitivity syndrome

## Footnotes

1 In this study, the term “science” refers to any branch of natural and social science.

2 The more recent term “Differences [or Disorders] of Sex Development” (DSD) also includes several sex chromosome variants that are not considered intersex, such as Turner or Klinefelter syndrome [11], which is why it will not be used in the context of this paper.
